# An Electrical Analogy Relating the Atlantic Multidecadal Oscillation to the Atlantic Meridional Overturning Circulation

**DOI:** 10.1371/journal.pone.0100306

**Published:** 2014-06-18

**Authors:** Bruce E. Kurtz

**Affiliations:** Independent consultant, Bradenton, Florida, United States of America; University of Vigo, Spain

## Abstract

The Atlantic meridional overturning circulation (AMOC) is the northward flow of surface water to subpolar latitudes where deepwater is formed, balanced by southward abyssal flow and upwelling in the vicinity of the Southern Ocean. It is generally accepted that AMOC flow oscillates with a period of 60–80 years, creating a regular variation in North Atlantic sea surface temperature known as the Atlantic multidecadal oscillation (AMO). This article attempts to answer two questions: how is the AMOC driven and why does it oscillate? Using methods commonly employed by chemical engineers for analyzing processes involving flowing liquids, apparently not previously applied to trying to understand the AMOC, an equation is developed for AMOC flow as a function of the meridional density gradient or the corresponding temperature gradient. The equation is based on the similarity between the AMOC and an industrial thermosyphon loop cooler, which circulates a heat transfer liquid without using a mechanical pump. Extending this equation with an analogy between the flow of heat and electricity explains why the AMOC flow oscillates and what determines its period. Calculated values for AMOC flow and AMO oscillation period are in good agreement with measured values.

## Introduction

The Atlantic multidecadal oscillation (AMO) is the regular variation in North Atlantic sea surface temperature (SST) with a period of 60–80 years first formally recognized (but not named) in 1994 by *Schlesinger and Ramankutty* [1] using spectrum analysis of temperature records from 1858–1992. *Kerr* [2] first used the term AMO in a 2000 review article, linking it to warming beginning around 1910 and peaking around 1940 followed by cooling ending in the mid-′70s. *Delworth and Mann* [3] reported a temperature oscillation with a period of approximately 70 years using proxy-based reconstructions of surface temperatures during the past 330 years and suggested fluctuations in the Atlantic meridional overturning circulation (AMOC) as the cause. *Enfield et al.* [4] in 2001 reported a 65–80 year cycle in North Atlantic SST data for the years 1856–1999. *Gray et al.* [5] in 2004 created a tree-ring based reconstruction of the AMO going back to 1567 with a 60–100 year cycle and tied this to SST anomalies from *Kaplan et al.* [6]. In 2005 *Sutton and Hodson* [7] showed evidence for the AMO using an index based on annual mean SST observations and *Polyakov et al.* [8]–[9] reported a 50–80 year ocean surface temperature cycle that is exceptionally strong in the Arctic. *Knudsen et al.* [10] in 2011 used climate proxy data to suggest that a 55–70 year AMO has persisted over at least the last 8000 years. Knight et al. [11]–[12] and Wei et al. [13] claimed that the AMO is internally driven rather than externally forced. Hu et al. [14], Wang et al. [15], Kumar et al. [16] and Edwards et al. [17] noted the important influence of the AMO on regional and global climate. It has been observed that the increase in Northern Hemisphere surface temperature over the past century has not been monotonic [18] and the AMO is believed to contribute to this variability [19]–[21].

It is generally accepted that the AMO is a result of regular variation in the AMOC [22]–[23]. The AMOC is sometimes referred to as the thermohaline circulation (THC), a term that will not be used here because of its imprecise meaning [24]–[25]. This circulation was first described in 1959–61 by Stommel and Arons [26]–[27], who proposed a global circulation comprising northward flow in the upper layer of the Atlantic Ocean ending in deepwater formation in the subpolar region, balanced by southward abyssal return flow to upwelling in the vicinity of the Southern Ocean. The AMOC is the primary means for transporting heat from the Atlantic equatorial region to northern latitudes [28].

Deepwater formation, the sinking of surface water due to cooling and ice formation increasing seawater salinity and density, was initially emphasized as the primary driver of the AMOC [29], but Kuhlbrodt et al. [30] in 2007 stated, “It is evident that surface buoyancy fluxes cannot provide the energy that is necessary to drive the AMOC.” The problems in understanding how the AMOC is driven have been addressed by incorporating other processes in addition to deepwater formation, including ocean eddy fields and the influence of wind stress on overturning [31]. This has resulted in proposed AMOC circulation mechanisms of great complexity: driven by the surface buoyancy flux in the North Atlantic and by energy from internal diffusion in the upwelling branch and upper-ocean Southern Ocean to South Atlantic transport, all influenced by winds, basin geometry, bottom topography, advection, and small-scale processes [32].

Contrasting with this complexity is the idea that the AMOC is driven primarily by the meridional density gradient. This seems to have begun with Marotzke et al. in 1988 [33] who stated that circulation is driven by the density difference between equatorial and polar seawater. Rahmstorf in 1996 [34] claimed that the overturning circulation behaves as if driven by the Atlantic Ocean density gradient with flow having a linear dependency on density difference. Using an ocean general circulation model, Marotzke [35] indicated that the strength of the meridional overturning circulation is not much affected by wind forcing, except very near the surface. Straneo [36] mentioned that the loss of heat in northern latitudes creates meridional density gradients linked to the AMOC. Schewe and Levermann [37] indicated that meridional density differences have been assumed to be key to determining the overturning rate of the AMOC and listed several supporting references. Wang et al. [38] suggested that the AMOC flow depends on the density gradient between the subpolar and subtropical North Atlantic.

An equation relating AMOC volumetric flow to density gradient is derived here that gives results in good agreement with measured AMOC flows. This equation is extended to relate AMOC heat flow to temperature gradient that is combined through an electrical analogy with AMOC thermal capacitance to give an equation for AMOC oscillation period that gives results in good agreement with observed AMO periods.

## Methods

The methods used to understand how the meridional density gradient drives the AMOC will be familiar to chemical engineers because, in the chemical process industry, change in liquid density with temperature is often used as a means for inducing liquid circulation. These methods seem not to have been previously applied to analyzing how the AMOC is driven. It is best to begin with a description of an industrial apparatus that resembles the AMOC: the thermosyphon loop cooler.

### 1. The Thermosyphon Loop Cooler and the AMOC

**Figure 1:**
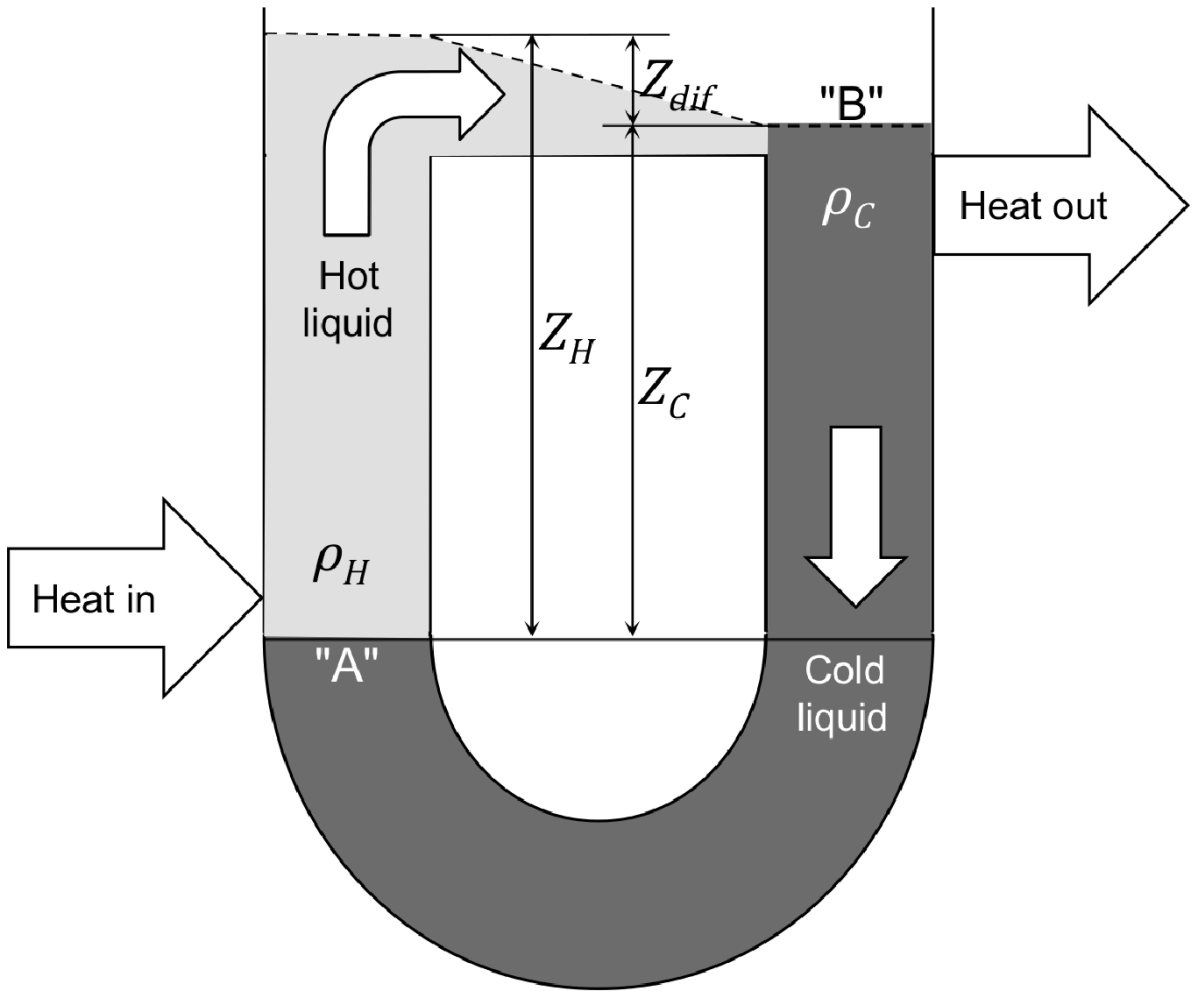
Thermosyphon loop cooler. This apparatus is used in industrial applications for advective transport of heat from source to sink with a circulating liquid without employing a mechanical pump. Heat is added to the left leg and removed from the right leg. The density in the hot leg (*ρ_H_*) is lower than in the cold leg (*ρ_C_*), so the liquid level in the hot leg (*Z_H_*) must be higher than in the cold leg (*Z_C_*) to maintain the force balance between the two legs. The resulting difference in level (*Z_dif_*) causes the flow to circulate. The apparatus is self-regulating because an increase in heat input increases the temperature of the hot liquid, which decreases its density, increases the difference in level between the two legs, increases the flow rate, and thus increases the rate of heat removal. The thermosyphon loop cooler behaves in basically the same way as the AMOC.

The thermosyphon loop cooler is used in industrial applications where heat is to be transferred with a circulating liquid in situations where a mechanical pump should not be used because of the unacceptable consequences of pump failure or because of corrosive/hazardous properties of the circulating liquid. As shown by Fig. 1, the thermosyphon loop cooler in its simplest form is a U-tube with a partially filled pipe connecting the tops of the two legs. Heat is added to the left leg and removed from the right leg, usually by way of integral heat exchangers (not shown). In some applications the heat source is an *in situ* exothermic chemical reaction and the circulating liquid is the reaction medium. Since the density in the hot leg (*ρ_H_*) is lower than in the cold leg (*ρ_C_*), the liquid level in the hot leg (*Z_H_*) must be higher than in the cold leg (*Z_C_*) to maintain the force balance between the two legs. The resulting difference in level (*Z_dif_*) creates the driving force that causes the flow to circulate. The balance between the driving force and the opposing force created by friction determines the steady-state circulating flow rate, so the point of heat input “A” is located as close to the bottom of the U-tube as practical and the point of heat output “B” as close to the top of the U-tube as practical to maximize the difference in level, and the cross-sectional area normal to the flow is made as large as practical to minimize the frictional force. The thermosyphon loop cooler is self-regulating because increasing heat addition increases hot leg temperature, decreases liquid density, increases difference in head (*Z_dif_*), and increases the flow of liquid (and the rate of heat transport) to the cold leg.

The thermosyphon loop cooler is like the AMOC in several respects: the hot leg corresponds to the tropical Atlantic with “A” being the bottom of the thermocline, the cold leg corresponds to the North Atlantic deepwater formation region with “B” being the ocean surface, and the connecting partially-filled pipe corresponds to the North Atlantic AMOC surface flow. But, in contrast to the thermosyphon loop cooler, with the AMOC the bottom of the thermocline (a few hundred meters deep) is far above the bottom of the recirculation path (a few thousand meters deep). This is contrary to Sandström’s 1908 argument [39] that thermally-induced circulation requires the heat source to be located below the cooling source at a depth close to the bottom of the recirculation path, but Jeffreys [40] in 1925 rebutted that argument by showing how circulation can be maintained through temperature difference irrespective of the relative heights of the heat source and sink, using the action of a thermosyphon loop as an illustration.

Another difference between the thermosyphon loop and the AMOC is that the upwelling connecting the return flow from the cold leg to the hot leg is not directly beneath the hot leg but many thousands of kilometers away in the Southern Ocean. This can happen because frictional resistance opposing return-flow through the deep ocean is relatively much smaller for the AMOC than for an industrial thermosyphon loop cooler since the ocean cross-sectional area normal to the flow is very large. The deep ocean acts as a reservoir, not a defined flow path, which is consistent with the lack of observational evidence for a direct connection between deepwater formation and abyssal return flow [41]. For the AMOC, the down-flow created by deepwater formation in subpolar northern latitudes is balanced by a corresponding up-flow at a location where the vertical temperature gradient is least supportive of density stratification: the Southern Ocean. The cross-sectional area normal to the up-flow is again very large, so the resistance to flow is very small.

All that is required to close the AMOC circulation loop is a means for conveying seawater from the Southern Ocean to the tropical Atlantic. This may be accomplished by the meridional component of the Atlantic circumpolar current (ACC). The ACC is driven by wind shear from the strong westerly winds in the latitudes of the Southern Ocean, causing surface water to flow northward via Ekman transport resulting from the Coriolis force. The ACC flow rate is about 140 Sv [42]–[43] (one Sv = 10^6^ m^3^s^−1^), much larger than the AMOC flow rate. Marshall and Radko [44] use residual-mean theory to describe how the ACC establishes meridional circulation and Rintoul et al. [45] discuss the relationship between the ACC and meridional circulation.

Deepwater formation, although unable to drive the AMOC by itself, is a necessary condition for the existence of the AMOC since it is essential to the existence of abyssal return flow. The absence of significant overturning in the Pacific Ocean is consistent with the absence of extensive deepwater formation in that ocean.

In contrast to the thermosyphon cooler, excess flow from any AMOC step can be diverted away from the succeeding step into the ocean proper, so the capacity for deepwater formation only needs to be equal to or greater than the rate at which the AMOC North Atlantic surface flow delivers seawater to the region of deepwater formation. This loose coupling among the three means that the slowest of the steps controls the rate of the overall process; so it is only necessary to understand the rate-controlling step in order to understand the behavior of the AMOC.

### 2. The Rate-controlling Step for the AMOC

**Figure 2:**
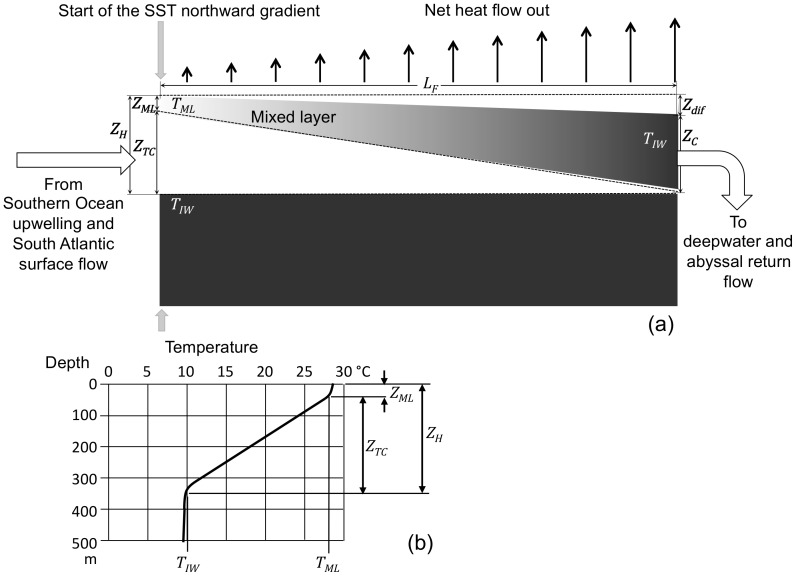
AMOC North Atlantic surface flow. (a) Darker color represents higher density. The hot (south) end of the flow path is defined (imprecisely) as the latitude where the SST northward gradient starts. The height of seawater above the bottom of the thermocline point (*Z_H_*) is the combined depth of the mixed layer (*Z_ML_*) and the thermocline (*Z_TC_*). The cold (north) end of the flow path is defined as the latitude where the mixed layer temperature becomes equal to the temperature at the bottom of the thermocline (*T_IW_*). At the cold end of the flow path the height of seawater (*Z_C_*) is defined as the vertical distance from the sea surface to the bottom of the thermocline at the hot end. The driving force for flow is the difference in sea surface heights between the two ends of the flow path (*Z_dif_*). The vertical arrows represent the net heat flow out from the ocean surface due to radiative cooling, increasing with increasing latitude. (b) Typical tropical Atlantic temperature-depth profile representing the south end of the AMOC flow path. The temperature of the mixed layer at that point is nearly uniform down to the upper inflection point (*T_ML_*) and the temperature of the thermocline decreases linearly with depth down to the lower inflection point, marking the beginning of intermediate water (*T_IW_*).

Figure 2 (a) shows how the density varies along the length of the AMOC flow path (*L_F_*), which begins in the tropical Atlantic at the latitude corresponding to the beginning of the northward SST gradient slightly north of the equator. As shown by the temperature-depth curve of Fig. 2 (b), the upper temperature inflection defines the depth of the mixed layer (*Z_ML_*) and the mixed layer temperature (*T_ML_*), and the lower temperature inflection defines the depth of the thermocline (*Z_TC_*) and the intermediate water temperature (*T_IW_*). The term “intermediate water” is used to identify the bottom of the thermocline and should not be confused with the same term when used to describe ocean water masses in other contexts. The mixed layer temperature is nearly constant with depth (within 0.5°C of the SST) while the thermocline temperature changes from *T_ML_* to *T_IW_* in a nearly straight line with increasing depth. Below the bottom of the thermocline the slope of the temperature-depth curve is much smaller and gradually approaches deepwater temperature (2–3°C). The AMOC flow is driven by the difference in sea surface heights between the two ends of the flow path (*Z_dif_*).

The horizontal temperature and density gradients are at a maximum in the ocean mixed layer and decrease with increasing depth through the thermocline, becoming zero at the bottom of the thermocline. Moving north along the flow path, *T_ML_* decreases while *T_IW_* remains constant. The point at which *T_ML_* equals *T_IW_* defines the end of the flow path for the present purpose. The extension of the horizontal density gradient beyond that point as the SST continues to fall below *T_IW_*, eventually reaching the temperature of deepwater formation, is not relevant to the development of the equation for AMOC flow near the south end of the flow path.

### 3. Equations for AMOC Flow and Period: Notation

Named variables and constants that are used in multiple instances in the derivation of the equations for AMOC flow and oscillation period are listed here for easy reference.

**Linear dimensions (See** **Fig. 2** **).**

*Z_H_*Depth to the bottom of the thermocline at the flow path hot (south) end.

*Z_C_*Depth to the *Z_H_* reference level at the flow path cold (north) end.

*Z_dif_*Difference between *Z_H_* and *Z_C_*.

*Z_ML_*Depth of the mixed layer at the flow path hot end.

*Z_TC_*Depth of the thermocline at the flow path hot end.

*Z_F_*Depth of flow path with vertically uniform density at the flow path hot end (calculated).

*L_F_*Length of the flow path.

*W_F_*Width of the flow path.

**Temperatures (See** **Fig. 2** **).**

*T_H_*Temperature (average) at the flow path hot end corresponding to *Z_H_*.

*T_IW_*Temperature at the flow path cold end and the thermocline bottom (intermediate water).

*T_dif_*Difference between *T_H_* and *T_IW_*.

*T_ML_*Temperature of the mixed layer corresponding to *Z_ML_*.

**Physical properties.**

*ρ_H_* Density (average) of seawater at the hot end of the flow path.

*ρ_C_* Density of seawater at the cold end of the flow path.

*ρ* Density of seawater averaged over the length of the flow path.

*µ* Viscosity of seawater averaged over the length of the flow path.

*C_p_* Specific heat of seawater averaged over the length of the flow path.

**Pressure, force, velocity and flows.**

*p_dif_* Pressure difference equivalent to *Z_dif_*.

*F* Force driving flow from density gradient.

*F'* Force opposing flow from friction.

*v* Average velocity across the flow path cross-section.

*Ψ* AMOC volumetric flow.

*q_IW_* AMOC heat flow based on *T_IW_* reference temperature.

**Other terms.**

*f_F_* Fanning friction factor.

*α* Ratio of seawater meridional density gradient to meridional temperature gradient.

*ΔH* Change in AMOC heat content per unit volume over the length of the flow path.

*G* Thermal conductance (advective) of the AMOC volumetric flow.

*C* Thermal capacitance of the AMOC heat storage layer.

*K* Coefficient in the exponential excitation function.

*s* Frequency in the exponential excitation function.

*t* Time in the exponential excitation function.

*T-* Period of oscillation.

### 4. Equation for AMOC Flow

The steady-state AMOC flow must balance the driving force (a function of meridional density gradient) against the opposing frictional force (a function of velocity). The seawater densities at the hot and cold ends of the flow path (*ρ_H_* and *ρ_C_*) and the length of the flow path (*L_F_*) determine the density gradient:
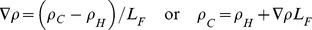



The vertical force balance requires that
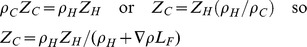



The difference between *Z_H_* and *Z_C_* is
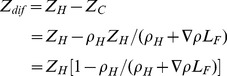
(1)


Converting *Z_dif_* to pressure difference, using *ρ* for the average density over the length of the flow path, gives
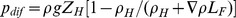



The force from the meridional density gradient that drives AMOC flow is the product of the pressure difference and the cross-sectional area normal to the direction of flow. The width of the flow path (*W_F_*) is bounded by the ocean width, but there is no physical boundary separating moving from stationary liquid that can be used to define the depth of the flow path. The development of the equation for the force opposing flow to follow assumes uniform flow over the cross-sectional area (vertically and horizontally), which is different from the actual situation for the AMOC where the horizontal density gradient driving flow is a maximum within the mixed layer (*Z_ML_*) and declines to zero with increasing depth through the thermocline (*Z_TC_*). To correct for this it is necessary to calculate a depth (*Z_F_*) for a flow path having vertically uniform density that exerts the same horizontal force as the actual mixed layer plus thermocline. Rearranging Eq. 1 and substituting mixed layer (*ρ_ML_*) and intermediate water densities (*ρ_IW_*) for the corresponding hot and cold densities gives:
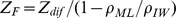



The value of *Z_F_* will be smaller than that of *Z_H_* (where *Z_H_* = *Z_ML_*+*Z_TC_*) depending on the relative depths of the mixed layer and thermocline (*Z_ML_* and *Z_TC_*). The force driving AMOC flow is then:
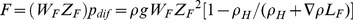
(2)


The frictional force opposing flow is proportional to the area of the wetted surface separating the moving and stationary liquid and the kinetic energy per unit volume of the liquid (Bird, Stewart and Lightfoot [46]). For a flow path of width *W_F_* and length *L_F_* the area of the wetted surface is *W_F_L_F_*. The kinetic energy term is *½ ρv^2^*. The proportionality factor relating these terms to the actual force is the Fanning friction factor, *f_F_*.
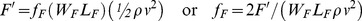
(3)


This equation is not a law of fluid dynamics; its only purpose is to define the friction factor. An alternative to the use of Eq. 2 for defining the friction factor is the Darcy-Weisbach equation, a phenomenological equation having the same form. The result is the Darcy (or Moody) friction factor (used by mechanical and civil engineers), which is four times larger than the Fanning friction factor (used by chemical engineers), a frequent source of confusion.

For laminar flow, the Hagen-Poiseuille equation, which is an exact solution of the Navier-Stokes equations, relates the friction factor to the Reynolds number. The AMOC is in laminar flow, even though the ocean is usually referred to as being *turbulent*. It would be better described as *agitated*. With *turbulent* flow the velocity fluctuates chaotically with time at every point throughout the volume of liquid and the energy causing this is derived directly from the flowing liquid. With *agitation* the energy creating chaotic velocity fluctuations is injected into the liquid from an external source (a turbine or propeller for industrial processes, wind shear for oceans) and the effects of this energy injection diminish in proportion to the distance from the energy source. Most of the energy injected into the ocean by wind shear is dissipated within the Ekman layer, a depth of only 10–20 m. From the Hagen-Poiseuille equation:
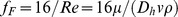



In the above equation *D_h_* is the hydraulic diameter, a parameter that allows the equation to be applied to flow cross-sections that are not circular. The definition of hydraulic diameter is *D_h_* = *4A_cx_/P_ws_* where *A_cx_* is the cross-sectional area normal to the direction of flow and *P_ws_* is the perimeter of the wetted surface.

Since *A_cx_* = *W_F_ Z_F_* and, for *W_F_*≫*Z_F_*, *P_ws_* = *W_F_*, then *D_h_* = *4Z_F_* and the expression for the friction factor becomes
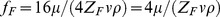
(4)


Combining Eqs. 3 and 4 gives the equation for the opposing force:
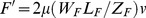
(5)


The equation for volumetric flow can now be obtained from the balance between driving and opposing forces, *F'* = *F*. Setting Eqs. 2 and 5 equal to each other and solving for velocity gives:
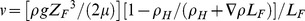
(6)


Multiplying velocity by the flow cross-sectional area (*W_F_ Z_F_*) gives the equation for AMOC volumetric flow:
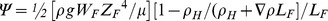



Since *ρ_H_*≫*∇ρ L_F_* and *ρ_H_*≅*ρ*, the equation for volumetric flow simplifies to
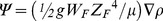
(7)


### 5. Equation for AMOC Oscillation Period

The equation for AMOC period derived in this section depends on the analogy between heat and electrical flows. The first step in developing the equation is to convert the expression for AMOC volumetric flow as a function of density gradient to an expression for AMOC heat flow as a function of temperature gradient. Assuming an approximately linear inverse relationship between temperature and seawater density over the temperature range of interest gives Eq. 8, where *α* is the ratio of density gradient to temperature gradient, *∇ρ/∇T*,
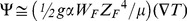
(8)


Equation 8 is multiplied by the change in heat content per unit volume, *ΔH* = *ρ C_p_* (*T_H_* –*T_IW_*), to obtain AMOC heat flow. Substituting the difference in temperature between the two ends of the flow path divided by the length of the flow path (*T_dif/_L_F_*) for *∇T*, gives Eq. 9, where *G* is the advective thermal conductance of the AMOC. Since *q_IW_* = *Ψ ΔH*, values for *q_IW_* can be obtained directly from measured values for *Ψ*.
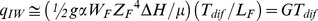
(9a)

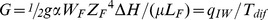
(9b)


Notice that heat flow equals conductance times the temperature difference, which is Ohm’s law. For *steady-state* AMOC heat flow this is a trivial result, but it suggests the possibility of an electrical analogy for describing the *dynamic* behavior of the AMOC flow. This is influenced not just by the meridional temperature difference (voltage) and opposing frictional force (resistance) but also by time-dependent variation in the heat stored by the ocean upper layer (capacitance). The thermal capacitance of the AMOC is equal to the product of seawater density, specific heat and the volume defined by the width and length of the flow path and the combined depth of the mixed layer and thermocline:
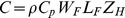
(10)


The electrical analog to the AMOC is a resistor and capacitor connected in series so the current (heat) flow through the resistor equals that through the capacitor. At time zero (south end of the flow path) the capacitor is fully charged. Increasing time (movement northward along the AMOC flow path) corresponds to decreasing charge in the capacitor (loss of heat by radiative cooling). When the capacitor is fully discharged (north end of the flow path) the total current (heat) flow through the capacitor, which is capacitance times the rate of change in voltage (temperature) with time, is equal to the total current (heat) flow through the resistor:
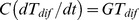
(11)


Substituting the exponential excitation function for the relationship between voltage (temperature) and time, *T_dif_* = *K e^st^* where *t* is time and *s* is the complex-frequency variable (called this because it can assume real, imaginary or complex values and has dimensions of reciprocal time) [47], and solving the resulting differential equation gives
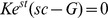



This equation is true for either *K* = *0* or *sC* = *G.* The first case corresponds to an initially uncharged state. The second case is
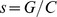



This is the *natural frequency* of the circuit (radians per second), the unforced frequency at which it will oscillate in response to a disturbance. It will also be the natural frequency of the AMOC. The corresponding *natural period* (seconds) is
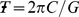
(12)


Equations 7 and 12 together enable calculation of AMOC flow and oscillation period. What remains is to test the results obtained from these equations against observed values.

## Results and Discussion

### 1. Independent Variables

The independent variables that determine AMOC flow in Eq. 7 are: the density gradient (*∇ρ*), flow path width (*W_F_*) and depth to the bottom of the thermocline (*Z_H_* = *Z_ML_*+*Z_TC_*). The density gradient is a function of the difference in densities between the two ends of the flow path and the length of the flow path (*L_F_*). The density at the south end of the flow path (*ρ_H_*) is a function of *T_ML_*, *T_IW_*, *Z_ML_*, and *Z_TC_*. This is calculated by assuming a linear temperature profile for the thermocline between *T_ML_* at depth *Z_ML_* and *T_IW_* at depth *Z_H_* and determining seawater densities (constant salinity) at five intermediate temperature points using a six-term polynomial approximation of the UNESCO equation of state for seawater. This is combined with the values of *Z_ML_* and *Z_TC_* to calculate a depth-weighted hot-end average density (*ρ_H_*). The density at the cold end of the flow path (*ρ_C_*) is determined from the temperature at the bottom of the thermocline (*T_IW_*), which applies over the full depth of the flow path at that location.

So the calculated values for AMOC flow (*Ψ*) and oscillation period (*T-*) are determined by six independent variables: temperatures of the mixed layer and intermediate water at the base of the thermocline (*T_ML_* and *T_IW_*), depth of the mixed layer (*Z_ML_*), depth to the bottom of the thermocline (*Z_H_*), and the length and width of the flow path (*L_F_* and *W_F_*). Values (or ranges) for the independent variables are:

*T_ML_* = 26 to 32°C

*T_IW_* = 10°C, from Fig. 2 (b)

*Z_ML_* = 60 to 140 m, from Chu & Fan [48]

*Z_H_* = 300 to 400 m, from Fig. 2 (b)

The values for the preceding variables are based on measurements and are reasonably certain. But the values for flow path length and width are fuzzy since the actual flow path is very complex and prone to wandering. About all that can be demanded is that the values be consistent with measured distances between the pertinent SST isotherms and the physical width of the North Atlantic. With values set for the first four independent variables (*T_ML_*, *T_IW_*, *Z_ML_*, and *Z_H_*) along with the desired values for AMOC flow and period (*Ψ* and *T-*), then finding a value for either flow path length (*L_F_*) or width (*W_F_*) consistent with that result determines the required value for the other.

**Figure 3:**
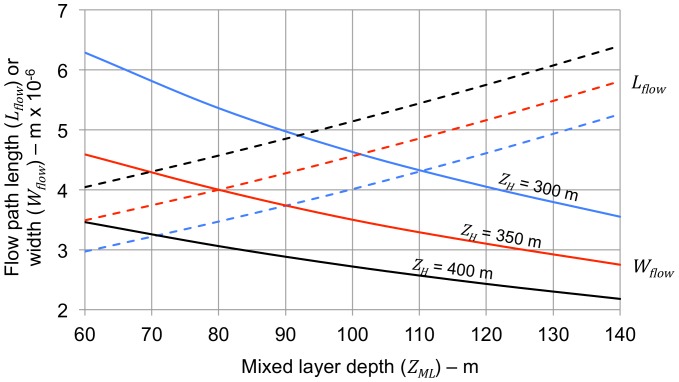
Values for flow path length and width to give a calculated AMOC flow of 17 Sv and oscillation period of 65 years. Mixed layer and intermediate water temperatures (*T_ML_* and *T_IW_*) are set at 28 and 10°C. Mixed layer depth (*Z_ML_*) varies from 60 to 140 m and depth to the bottom of the thermocline (*Z_H_*) is set at 300, 350, and 400 m (blue, red and black lines). Dashed lines are flow path length (*L_F_*); solid lines are flow path width (*W_F_*). Increasing mixed layer depth corresponds to increasing flow path length combined with decreasing flow path width to give a calculated AMOC flow (*Ψ*) and oscillation period (*T-*) of 17 Sv and 65 years. There is no practical way of accurately determining actual values for either flow path length or width, but the values shown are reasonably consistent with distances between the North Atlantic SST isotherms corresponding to the mixed layer and intermediate water temperatures, and with ocean widths.

A reasonable location for the hot (south) end of the AMOC path is just north of the equator where the northward temperature gradient begins, around 10 to 20°N. This is not far from the 26.5°N location where AMOC flow is being monitored. The equator itself is not a suitable location for the hot end of the flow path because the meridional temperature gradient at that point is essentially zero. A first estimate for path length (*L_F_*) is the distance from the hot end of the flow path to the location of the SST isotherm corresponding to the temperature at the base of the hot-end thermocline (10°C), around 50 to 60°N, based on SST profiles (Space Science and Engineering Center, University of Wisconsin-Madison, http://www.ssec.wisc.edu/data/sst/). Considering seasonal variation, the latitudinal wandering of the isotherms, and the sinuous nature of the AMOC flow, only very rough estimates of path length can be made. With the four independent variables other than path length and width fixed and the desired values for AMOC flow and period chosen, selecting a value for path length also fixes the value for path width (*W_F_*). This is illustrated by Fig. 3, showing values for flow path length and width consistent with values for AMOC flow (*Ψ*) and period (*T-*) of 17 Sv and 65 years as a function of mixed layer depth (*Z_ML_*) for different values of depth to the bottom of the thermocline (*Z_H_*). The path widths required for an AMOC flow of 17 SV and period of 65 years change in the opposite direction from the required path length with increasing mixed layer depth.

### 2. Reality Checks

As a first reality check, Eq. 1 is used to calculate the difference in head (*Z_dif_*) between the two ends of the flow path. Variation in ocean surface height due to wind-driven (geostrophic) flows is less than two meters by satellite altimetry, so *Z_dif_* must be smaller than this measured variation in ocean surface height but not so small as to be lost in the noise. The calculated values for *Z_dif_* range between 0.4 and 0.7 m over the chosen range of values for the independent variables, a reasonable result.

An independent reality check is to confirm that the power available from the AMOC as a heat engine is consistent with the pumping power required by the AMOC volumetric flow. All heat engines, natural or engineered, operate by adding heat from a *source* (solar heat gain) to a *working substance* (seawater) that is passed through a *working body* (the AMOC) to a heat *sink* (radiative heat loss). The working body converts part of this heat flow to power by exploiting certain properties of the working substance (change in seawater volume with temperature). The Malone engine [49]–[50], developed around 1925, created rotary motion by exploiting the change in water volume with temperature (it never attained commercial success). The pumping power required by the AMOC is the product of pressure difference and volumetric flow. A head of one-meter equals a pressure of about 10 kPa, so for an AMOC flow of 18 Sv the power required is about 0.2 TW, a tiny fraction of the AMOC heat flow of about 1200 TW. Therefore, the pumping power required by the AMOC flow can easily be extracted from its heat flow, even if it is a very inefficient heat engine.

There are two assumptions in the derivation of the AMOC flow equation that can be confirmed here as part of reality checking. The first is that the AMOC flow is laminar; the velocity (*v*) calculated from Eq. 6 gives a Reynolds numbers between 10 and 40, far down in the laminar range. The second is that the ratio of density gradient to temperature gradient (α) is approximately constant; the calculated value of α over a temperature range of 26–32°C is between −0.23 and −0.26, validating this assumption.

### 3. Calculated vs. Observed Values for AMOC Flow and Oscillation Period

**Figure 4:**
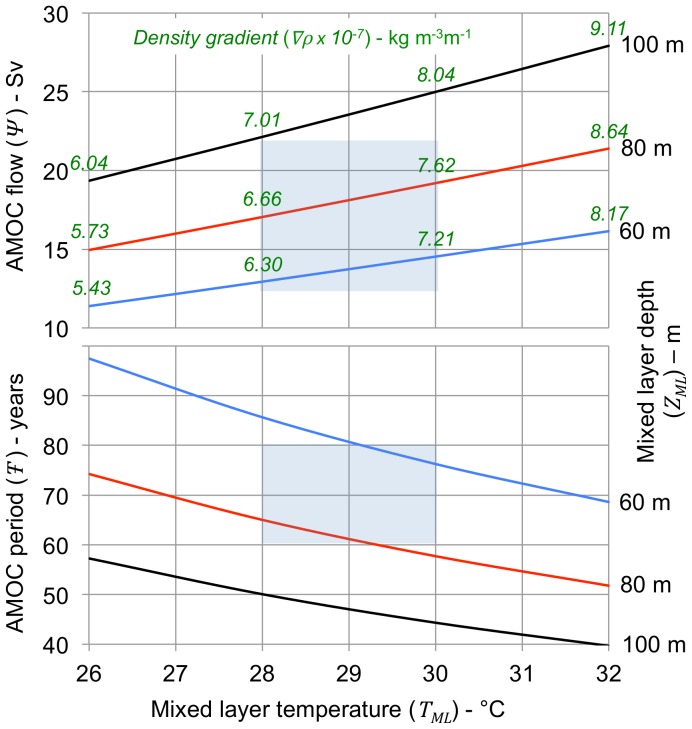
AMOC flow and period vs. mixed layer temperature. The curves show calculated AMOC volumetric flow (*Ψ*) and oscillation period (*T-*) for mixed layer temperatures at the south end of the flow path (*T_ML_*) ranging from 26 to 32°C with mixed layer depths (*Z_ML_*) of 60, 80, and 100 m (blue, red and black lines). The curves relating flow to mixed layer temperature show the values for density gradients (*∇ρ*) calculated from the equations. The depth to the bottom of the thermocline (*Z_H_*) is set at 350 m. The length of the flow path (*L_F_*) is set at 4000 km, roughly the annual average distance between the 28°C SST isotherm and the 10°C SST isotherm. The width of the flow path (*W_F_*) is set to the same value, consistent with Fig. 3. The shaded areas on the figures indicate the approximate range of observed values for AMOC flow (12–22 Sv) and oscillation period (60–80 years). Values for flow and oscillation period cannot be calculated independently; fixing one necessarily fixes the other.

The most important test of the derived equations is the comparison between calculated and observed values for AMOC volumetric flow and oscillation period. Figure 4 (upper) shows calculated AMOC flow (*Ψ*) as a function of the mixed layer temperature (*T_ML_*) for mixed layer depths (*Z_ML_*) of 60, 80 and 100 m. Flow path length (*L_F_*) and width (*W_F_*) are both set to 4 x10^6^ m and mixed layer temperature (*T_ML_*) is set to 10°C. Recent published values for flow from the RAPID AMOC observing system [51] located at 26.5°N are in the range of 12 to 22 Sv, consistent with calculated values. The flow rate is a function of the meridional density gradient (*∇ρ*), values for which are shown on the upper figure. The lower part of the figure shows the corresponding calculated values for the AMOC oscillation period (*T-*). Observed values for AMO periods are in the range of 60–80 years, also consistent with calculated values.

**Figure 5:**
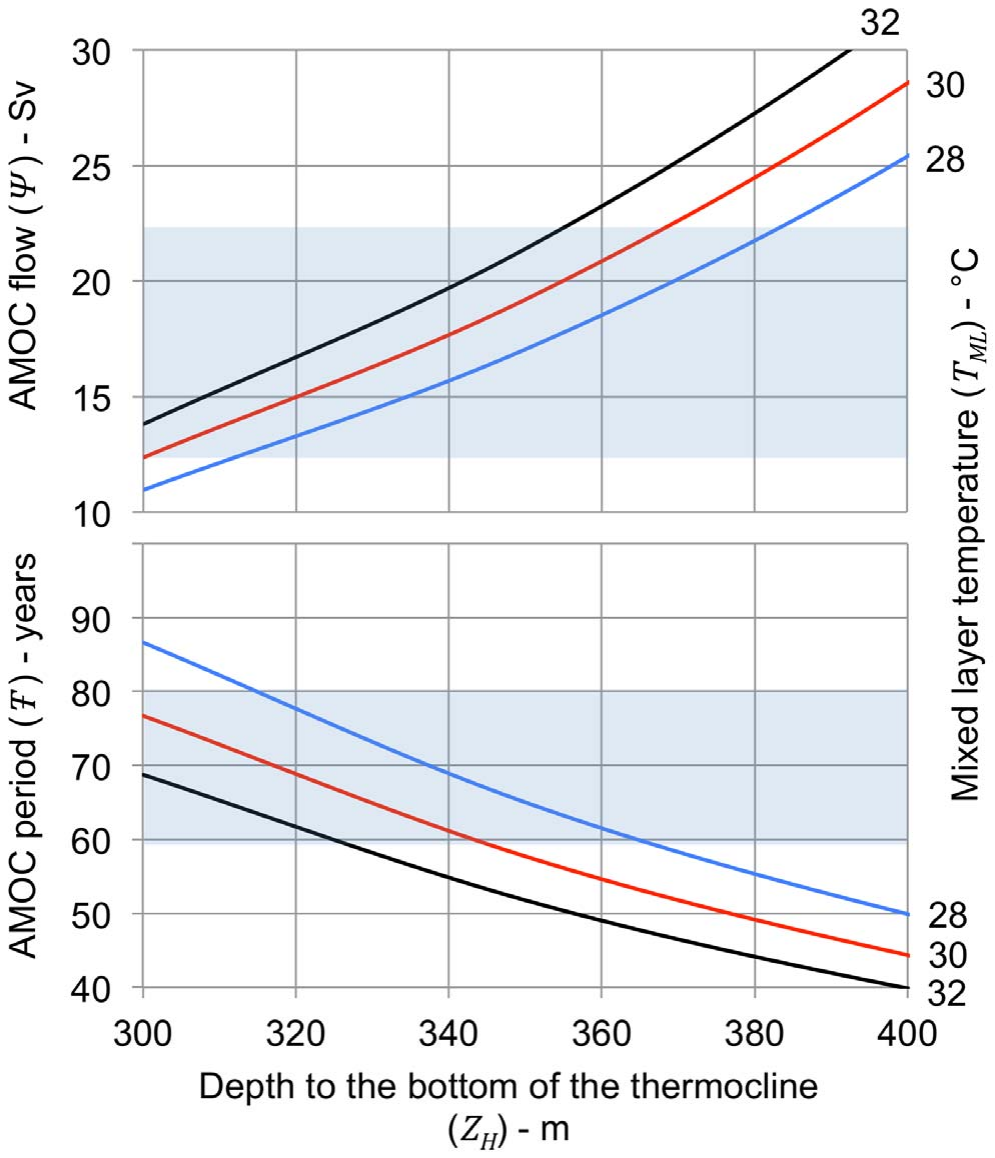
AMOC flow and period vs. depth to the bottom of the thermocline. The curves show calculated volumetric flow (*Ψ*) and oscillation period (*T-*) for depths to the bottom of the thermocline at the south end of the flow path (*Z_H_* = *Z_ML_*+*Z_TC_*) ranging from 300 to 400 m for mixed layer temperatures (*T_ML_*) of 28, 30, and 32°C (blue, red and black lines). The length and width of the flow path (*L_F_* and *W_F_*) are the same as for Fig. 4. The sensitivity of the calculated values for AMOC flow and period to the depth to the bottom of the thermocline is high because the depth of the flow path *Z_F_* used in the calculation of AMOC flow is related to the depth to the bottom of the thermocline, ranging between 0.5 and 0.7 of *Z_H_* depending on the relative depths of the mixed layer and thermocline. This term appears as *Z_F_^4^* in Eq. 7. The shaded areas on the figures indicate the approximate range of observed values for AMOC flow and oscillation period.

Figure 5 shows calculated AMOC flow (*Ψ*) and period (*T-*) as a function of depth to the bottom of the thermocline (*Z_hot_*) at the hot end of the flow path for mixed layer temperatures (*T_ML_*) of 28, 30 and 32°C. Flow path length (*L_F_*) and width (*W_F_*) are both set to 4 ×10^6^ m and mixed layer depth (*Z_ML_*) is set to 80 m.

### 4. Nature of the Equations

The equations are derived entirely from first principles: there is no empirical or phenomenological content nor any *ad hoc* factor that can be adjusted to improve the agreement between calculated and measured results. Once values for the equation independent variables are set, so are both AMOC flow and oscillation period since the dimensions of the flow path (depth, width and length) are common to the calculations of both these quantities. Judicious selection of values for the equation independent variables that lie comfortably within their range of uncertainty can result in remarkably good agreement between calculated and observed values for AMOC flow and oscillation period. Although supportive of the basic soundness of the equations, this should not be mistaken for an ability to accurately predict the behavior of the AMOC in the absence of observational data uncertainty that can be used for calibration of the independent variables.

Although the AMOC is generally seen as a very complex phenomenon, the density-driven flow described by the derived equations is not complex. The main contribution to the apparent AMOC complexity must be the superimposed wind-driven geostrophic flows. These are comparable in magnitude to AMOC density-driven flow, but evidently their *net* contribution to meridional flow is small. There are also secondary deep flows created by viscous drag, causing the actual velocity profile to extend beyond the bottom of the flow path depth used in the flow calculation and beyond the thermocline as well. Because of total energy constraints these secondary flows are not likely to have much effect on total AMOC volumetric flow.

The methods used here for deriving the equations are widely used by chemical engineers, but seem not to have been previously applied to the AMOC. Despite the simplifying assumptions implicit in the equations, especially the assumed confinement of the AMOC to the defined flow path and the neglect of horizontal or vertical velocity gradients, they do a very good job of predicting the behavior of the AMOC/AMO. But they may not have much applicability to other ocean flows since the AMOC may be uniquely simple in the way it is driven.

## Conclusions

### 1. The AMOC is Driven Directly by the Meridional Density Gradient and Indirectly by the Corresponding Temperature Gradient

The AMOC flow calculated from equations based on the meridional density gradient driving force agrees well with measured flow.

### 2. AMOC Flow Oscillates at its Natural Frequency to Create the AMO, which is an *Oscillation* that is a Permanent Feature of the Earth’s Climate System, not a Stochastic *Variability*

The natural oscillation frequency calculated from the advective thermal conductance of the AMOC and ocean upper layer thermal capacitance agrees well with observed AMO frequency.

### 3. The AMOC Flow Oscillation may be a Contributing Factor to the Non-monotonic Nature of Global Warming

AMOC flow oscillation may cause heat to accumulate in the Southern Hemisphere ocean when the flow is below its steady-state value (cool phase of the AMO), to be released via Northern Hemisphere radiative cooling when the flow is above its steady-state value (warm phase of the AMO), thus superimposing a sinusoidal temperature signal on monotonic global warming.
